# Non-*aureus* Staphylococci and Bovine Udder Health: Current Understanding and Knowledge Gaps

**DOI:** 10.3389/fvets.2021.658031

**Published:** 2021-04-15

**Authors:** Jeroen De Buck, Vivian Ha, Sohail Naushad, Diego B. Nobrega, Christopher Luby, John R. Middleton, Sarne De Vliegher, Herman W. Barkema

**Affiliations:** ^1^Department of Production Animal Health, Faculty of Veterinary Medicine, University of Calgary, Calgary, AB, Canada; ^2^Ottawa Laboratory Fallowfield, Canadian Food Inspection Agency, Ottawa, ON, Canada; ^3^Department of Large Animal Clinical Sciences, Western College of Veterinary Medicine, University of Saskatchewan, Saskatoon, SK, Canada; ^4^M-team and Mastitis and Milk Quality Research Unit, Department of Reproduction, Obstetrics and Herd Health, Faculty of Veterinary Medicine, Ghent University, Merelbeke, Belgium; ^5^Department of Veterinary Medicine and Surgery, College of Veterinary Medicine, University of Missouri, Columbia, MO, United States

**Keywords:** mastitis, bovine, *Staphylococcus*, mammary gland, udder, bacterial infection

## Abstract

Despite considerable efforts to control bovine mastitis and explain its causes, it remains the most costly and common disease of dairy cattle worldwide. The role and impact of non-*aureus* staphylococci (NAS) in udder health are not entirely understood. These Gram-positive bacteria have become the most frequently isolated group of bacteria in milk samples of dairy cows and are associated with (mild) clinical and subclinical mastitis. Different species and strains of NAS differ in their epidemiology, pathogenicity, virulence, ecology and host adaptation, and antimicrobial resistance profiles. They have distinct relationships with the microbiome composition of the udder and may also have protective effects against other mastitis pathogens. Some appear to persist on the skin and in the teat canal and udder, while others seem to be transient residents of the udder from the environment. Analyzing genotypic and phenotypic differences in individual species may also hold clues to why some appear more successful than others in colonizing the udder. Understanding species-level interactions within the microbiome and its interactions with host genetics will clarify the role of NAS in bovine mastitis and udder health.

## Introduction

Staphylococci can be subdivided into two groups, coagulase-positive and coagulase-negative, based on their ability to clot rabbit plasma, a key diagnostic step in clinical microbiology laboratories. Staphylococcal coagulase is an extracellular protein encoded by the *coa* gene. *Staphylococcus* coagulase-associated clotting involves formation of a coagulase-prothrombin complex that recognizes fibrinogen as a substrate and directly converts it into fibrin. Coagulase secretion is a key virulence strategy in pathogenesis and persistence of staphylococcal diseases (1) and has often been used to distinguish *S. aureus* from other staphylococci (2). In the context of bovine mastitis, staphylococci were historically classified into two groups: one that included *S. aureus*, considered more pathogenic and thus a “major pathogen,” and a second including other staphylococci that were lumped together as “minor pathogens” and termed the coagulase-negative staphylococci (CNS).

Another classification scheme adopted in more recent mastitis literature involves grouping all staphylococci other than *S. aureus* into a single category, non-*aureus* staphylococci (NAS). Some coagulase-positive and coagulase-variable mastitis pathogens (e.g., *Staphylococcus hyicus* and *Staphylococcus agnetis*) were also often included in the coagulase-negative category. In addition, coagulase-negative variants of *S. aureus* are known to exist, some of which can have similar pathogenicity to their coagulase-positive variants (3). Some *S. aureus* isolates of bovine origin react negatively to the standard coagulase test and are PCR-negative for the *coa* gene (4, 5). Additionally, the von Willebrand factor-binding protein exhibits coagulating ability, resulting in *S. aureus* producing two proteins that coagulate plasma (6). In a study analyzing the distribution of virulence factor genes among isolates belonging to 25 NAS species of bovine origin, the corresponding gene for the von Willebrand factor-binding protein, *vWbp* was detected in *S. agnetis, S. hyicus*, and *S. chromogenes* (7). Because the term coagulase-negative staphylococci, based on the ability of proteins to cause coagulation as a diagnostic test, may result in ambiguity in the context of mastitis, non-*aureus* staphylococci (NAS) provides a better term to classify pathogens associated with bovine mastitis by providing a clear dichotomy between *S. aureus* and the other staphylococcal species. Furthermore, NAS are often considered pathogens of lesser importance in dairy production (so-called minor pathogens), especially compared to *S. aureus*, some streptococci and some coliforms (8). However, in most studies they have been the most frequently isolated bacteria from udder quarters with subclinical mastitis (SCM) (9) and their ability to cause clinical mastitis (CM) cannot be understated. Approximately 20% of milk samples collected on Canadian dairy farms were NAS-positive and the prevalence of NAS in quarters with a somatic cell count (SCC) <200,000 cells/mL, oftentimes regarded as healthy udder quarters, was ~43% (10, 11), suggesting at least some can be considered commensals (12). In a Canada-wide clinical mastitis (CM) study (13), NAS were isolated from 10.7% of culture-positive samples, whereas in a CM study from Wisconsin (14), 6.1% of isolates were NAS. In two Belgian studies 5 and 12% quarters with CM, respectively, were NAS-positive (15, 16). Other studies in the US and Belgium also concluded that NAS are the principal cause of IMI on modern dairy farms (17, 18). Prevalence of IMI with NAS is especially high in virgin and first lactation heifers (18–24). In addition, it has been argued that modern mastitis control programs, which focus on major udder pathogens (and are apparently less effective against minor pathogens such as NAS), may have contributed to marked increases in prevalence of IMI due to NAS (23, 25). On dairy farms implementing modern mastitis control practices, the prevalence of major pathogen IMI has decreased resulting in a lower bulk tank SCC. NAS IMI have become relatively more important and are considered the leading cause of SCM (23).

NAS do not seem to be the main cause of mastitis in herds with significant milk quality problems (Table 1); yet, in herds with low bulk tank SCC, NAS IMIs contribute to a substantial proportion of the bulk tank SCC (8). However, a recent longitudinal study demonstrated that NAS IMI early in lactation results in only a small but significant increase of SCC (24), and other studies demonstrated that when compared to non-infected quarters, NAS-infected quarters did not generally have reduced milk production (26, 27). While their effect on milk yield at the whole cow level has no negative impact (28), NAS-infected heifers out-produced non-infected counterparts, presumably due to a lower incidence of CM (29, 30). One study reported a positive correlation of *S. caprae* with milk yield in goats, further suggesting that NAS IMI may have a positive effect in early lactation on milk yield (31, 32) yet cows with SCM produced milk of poorer quality (2). Elucidating factors to better understand the role of NAS in IMI (Table 1) may lead to more effective prevention and control measures of SCM.

**Table 1:** Knowledge gaps in understanding the role of NAS on udder health.

**Section**	**Knowledge gaps**
Species distribution and diversity	• Interactions between individual NAS (e.g., synergistic) in the udder • Interactions between individual NAS and the udder • Acquired genes giving the ability to colonize and persist in udders and on teat apices
Dominant NAS species	• Factors that underlie success of certain NAS as colonizers and the most prevalent species such as *S. chromogenes*
Impact of NAS on inflammation	• Potential strain differences and factors of NAS species that provoke inflammation
Virulence and host association	• Association between virulence genes and disease severity • The role of capsular genes in NAS virulence • Correlation between capsular genotype/phenotype and biofilm formation • Biofilm production and its association with pathogenicity of *S. chromogenes* and other NAS species • Elucidating the role of specific virulence factors (e.g., β-hemolysins) for *S. chromogenes* and other NAS species • Tracking evolutionary history of NAS species in the context of virulence genes
Antimicrobial resistance	• Clarifying if NAS species represent a reservoir of AMR genes for major mastitis pathogens • Possibility of new resistance mechanisms in NAS species • Characterization of intrinsic AMR mechanisms • Correlation between co-resistance profiles of NAS species and its effect on udder health
Niche adaptation and host association	• Classification of NAS species as commensal microbiota or opportunistic or obligate pathogens
Interactions within the udder microbiome	• Causes of NAS being disruptors of the udder microbiome • Role of bacteriocins produced by NAS species in modulating the udder microbiome • Clarifying if NAS species IMI increase susceptibility to major pathogens or, on the contrary, prevent them from infecting the udder • Characterizing the host genetic component and its relationship to NAS colonization
Understanding how mastitis control measures influence NAS incidence and prevalence	• Further evaluation of the associations among mastitis control measures and incidence and prevalence of mastitis caused by different NAS species

## Species Distribution and Diversity

Staphylococci have been isolated from many animal species. Very few of these NAS species (e.g*., S. hyicus, S. pseudointermedius, S. arlettae, S. felis, S. equorum, S. delphini*, and *S. caprae*) demonstrate a level of host specificity (32–37). NAS are very prevalent in bovine IMI, especially in dairy heifers (38, 39). In fact, 53 different species are recognized in the genus *Staphylococcus*, 23 of which have been isolated from a Canadian collection of > 5,000 bovine milk samples (9). Twenty-five species were identified from 300 samples in another study (40), whereas in a smaller study only 10 species were found from 105 NAS isolates (41).

**Figure 1:**
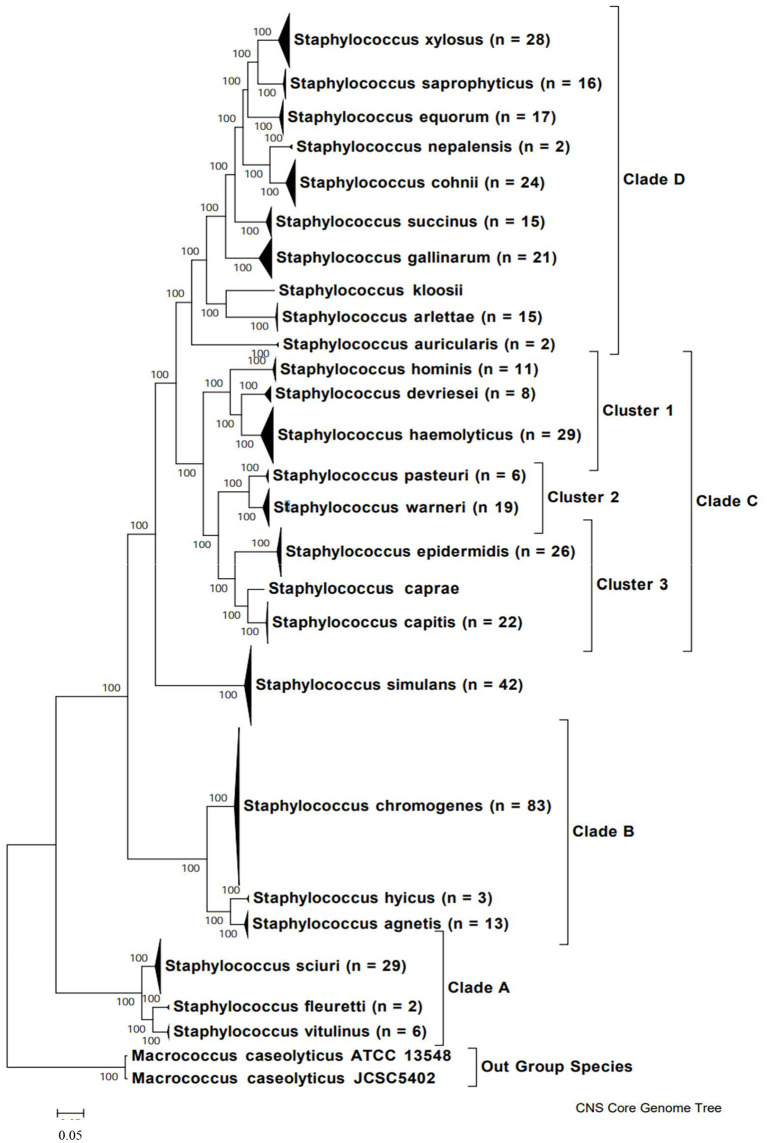
Phylogenetic tree of NAS species based on whole genome sequences, indicating major clades. Copied from Naushad et al. (43).

In order to understand the variety of NAS species isolated from milk, it is important to clearly determine phylogenetic relationships among species (Figure 1). Previously, this relationship was determined through construction of a phylogenetic tree based on 16S rDNA sequences of 42 NAS isolates (42). More recently, the genomes of over 400 bovine isolates were sequenced and several methods were applied to understand evolution and relationships between species (e.g., based on core protein set, entire genomes, SNPs). As a result, 5 main clades were identified, each with a varying number of species (43). Construction of a phylogenetic tree based on whole genome sequencing provided a highly reliable classification of bovine NAS species. Earlier studies using single gene sequencing revealed contradicting phylogenies when compared to each other, failing to show true evolutionary histories and speciation of *Staphylococcus*. By dividing bovine NAS species into 5 distinct clades, shared biological properties among related species such as virulence and host specificity can be better characterized. These properties will provide the basis for studies on the role and significance of individual and related NAS species for udder health, as there is also diversity within species isolated from different body sites on the same animal (44).

The diversity of NAS species begs the question of why so many *Staphylococcus* species can be isolated from bovine milk samples (Table 1). It is unclear if all NAS species fill the same niche and are therefore interchangeable; whether they are all unique in their interactions with the udder, or whether synergisms exist among species or strains. There are reasons to believe that a bacterial species only evolves to adapt to a certain niche and that every mutation in every gene needs to provide an advantage to be maintained. Following that reasoning, every NAS species, each with roughly 2.4 Mb genomes and many thousands genetic polymorphisms, must have vastly different behaviors. Besides, each species has a large pan genome, suggesting large strain differences within species. Analysis of NAS by Pulsed Field Gel Electrophoresis (PFGE) has demonstrated that diversity exists within species with respect to persistence and SCC in corresponding milk samples (45), and between isolates of the same species isolated from different body sites (44). This diversity suggests important differences in virulence and host adaptation genes, differences in gene expression between species, and differences in interactions with other microbes. Importantly, the identification methods and study designs in previous literature may have influenced these results. Two phenotypic tests (API Staph ID 32 and Staph-Zym) were shown to be inaccurate in species identification from bovine milk samples, and genotypic methods were shown to have higher type ability and accuracy in the identification of bovine NAS (46, 47). API Staph has been shown to have moderate to low performance in goat NAS identification as well (48). Moreover, biases in biochemical testing developed for human NAS should be considered in the context of characterizing species diversity and prevalence of bovine NAS as genotypic methods are considered more accurate than biochemical galleries (47). Additional large-scale longitudinal studies are needed to provide insight into how both strain and evolutionary differences affect prevalence and distribution of NAS species causing IMI, and the resulting impact on udder health. MALDI-TOF is an accurate technique in this regard, able to correctly identify almost all NAS isolates at the species-level, as long as the library is updated with relevant field isolates and strains from new species (49–51).

The ability of different NAS species to persist and colonize different niches may be due to acquired genes which confer selective advantages in their respective environment. For example, several factors, such as surface proteins, biofilm resistance genes, and phenol-soluble modulin peptides, increased the ability of *S. epidermidis* to persist in blood isolates obtained from newborn humans (52). Recently, the molecular relationship was determined between *S. agnetis* isolates from cattle and chickens. The chicken isolates were closely related to cattle isolates and clustered together, indicating a common ancestor and possibly a single jump from cattle to chickens (53). However, no unique virulence genes were identified in a hypervirulent chicken isolate, resulting in the speculation of small alterations in virulence associated factors.

## *Staphylococcus chromogenes*: The Dominant NAS Species

In a Canada-wide study, 50% of NAS isolates were *S. chromogenes* (Table 2). This NAS species had the highest prevalence in IMI of any bacteria in milk samples of cattle with SCM (and either a low or high SCC) (9, 38). *Staphylococcus chromogenes* was also the most prevalent species in a US study (55) and Belgian studies (56). In Canada, *S. chromogenes* also has the highest (of any NAS) prevalence in CM (as well in high or low SCC quarters). Of all NAS species, *S. chromogenes* (followed by *S. epidermidis*, and *S. simulans*)-positive milk samples had the highest SCC (23, 60–62). *S. chromogenes* IMI is associated with higher SCC and is considered an important species in quarters with a high SCC, persistent cases and CM (38, 45, 61, 63). It was also reported that *S. chromogenes* is responsible for significantly increased SCC in cows with persistent SCM (64), as well as for greater inflammatory responses and more pronounced clinical signs (65). NAS also play a major role in small ruminant mastitis. One study found that SCC increase was three times higher in small ruminant NAS than in bovine NAS IMI, and another reported an elicited immune response in goats after inoculation with *S. chromogenes* (66–68). This suggests that either the host immune response or differences in NAS must be taken into account when discussing bacterial virulence and commensalism.

**Table 2:** Overview of the top 3 most frequently isolated non-*aureus* staphylococci species in various countries from cows having subclinical or clinical mastitis.

**Country**	**Top 3 species**	**Prevalence (%)**	**Reference(s)**
Canada	*S. chromogenes S. simulans S. xylosus*	49 17 12	(9)
Belgium	*S. equorum S. haemolyticus S. epidermidis*	34 13 9	(40)
Finland	*S. chromogenes S. simulans S. warneri*	49 23 5	(54)
The Netherlands	*S. chromogenes S. epidermidis S. capitis*	30 13 11	(23)
United States of America	*S. chromogenes S. haemolyticus S. simulans*	48 18 7	(55)
Belgium	*S. chromogenes S. sciuri S. cohnii*	41 13 11	(56)
China	*S. arlettae S. sciuri S. xylosus*	12 12 12	(57)
Poland	*S. warneri S. chromogenes S. xylosus*	37 33 23	(58)
Argentina	*S. chromogenes S. haemolyticus S. warneri*	47 32 7	(59)
Belgium	*S. chromogenes S. haemolyticus S. equorum*	10 9 7	(16)
Belgium	*S. chromogenes S. xylosus S. vitulinus*	29 9 9	(27)

*Quarter milk samples were examined in each study, apart from one which used bulk tank samples (40). Samples were considered NAS-positive according to the National Mastitis Council guidelines, in addition to genotypic characterization of species*.

Interestingly, *S. chromogenes* is most frequently isolated from milk and skin (69), but not from other environmental sources, suggesting that it is likely host-adapted (49, 62, 70–72). Literature suggests that this species is largely isolated from samples of bovine origin, although it can be isolated from the milk of other dairy ruminants including goats and dairy buffalo (73). According to Taponen et al. (54), 55% of *S. chromogenes* persisted throughout lactation, while Fry et al. (45) showed persistence based on PFGE. Another study reported the average duration of IMI caused by *S. chromogenes* to be ~40 days longer than that of other species (28). Additionally, 45% IMI caused by *S. chromogenes* was shown to persist over at least two sampling days, compared to only 9.8% of other species persisting for that long (28). The average duration of *S. chromogenes* IMI was reported to be 150 d in another study (63). Moreover, infection by one *S. chromogenes* genotype, followed by recovery, then re-infection with a different *S. chromogenes* genotype may be misclassified as a chronic *S. chromogenes* IMI in the absence of strain-typing data. Although PFGE-based strain-typing of the first and last IMI isolates in a series from the same quarter (45) indicated persistence, the duration of *S. chromogenes* IMI and all other NAS species may therefore be overestimated by studies that have not included a strain-typing method.

## Other Prevalent NAS Species in Different Geographical Regions

Following *S. chromogenes*, the most frequently identified NAS are *S. simulans, S. xylosus, S. haemolyticus*, and *S. epidermidis*. While there are some regional differences in overall prevalence (Table 2), these species are consistently isolated from the udder and milk samples. In contrast, the other NAS species together represent <10% of the NAS isolates. While it might be concluded that regional and environmental differences affect the prevalence and distribution of individual NAS species (Table 2), it is also reasonable to conclude that species distribution is most likely impacted by herd management (69). Hence, regional differences are perhaps more impacted by the nature of the studied herds than geography. These findings suggest that additional studies are needed to better characterize these influences.

Interestingly, some *Staphylococcus* species are infrequently isolated from milk, e.g., *S. rostri* was isolated in one study from feces (16, 74). NAS species which can be isolated from other sites on the cow, but not from milk or the exterior of the udder might provide an opportunity to help clarify which genes allow NAS to either infect or colonize the udder.

## Association of IMI and Udder Inflammation for Different NAS Species

Host-pathogen interactions for many mastitis pathogens has not been well-established because of the complex interactions *in vivo*, and much of the evidence derived from the interactions of *S. aureus* with its host. It is of great interest to determine whether all NAS species provoke inflammation and increases in SCC. Most studies, evaluating the associations between mammary inflammation (e.g., SCC) and presence of NAS in milk samples have been observational. Some conflicting information on effect of NAS IMI on udder health (8, 63, 69) and the impact on milk yield (26, 29) exists within literature. Large scale studies using 16S, *rpoB* sequencing (9) and MALDI (50, 75) are targeting these questions. Interestingly, when comparing the prevalence of individual NAS species between milk samples with low SCC (<200,000 cells/mL) or high SCC (≥200,000 cells/mL), all species had higher prevalence in the latter, suggesting that NAS provoke some inflammatory response (38). In addition to an increase in SCC, NAS IMI was shown to have elicited host immune responses, an important consideration is the role of NAS in modulating these responses, as they may offer cross-protection against other mastitis pathogens (64, 65, 76, 77).

Few studies have evaluated NAS species and their influence on udder inflammation in experimental intramammary challenge trials (76, 77). Based on these studies, it was demonstrated that intramammary challenge with *S. chromogenes* stimulated an inflammatory response and that a strain previously isolated from an IMI was more inflammatory than a teat apex strain. Furthermore, while *S. fleurettii* could be isolated from milk of experimentally infected udder quarters and was associated with an increase in SCC, the strain was cleared from milk within 12 h (76). Hence, more data are needed to truly understand the relationship between IMI and udder inflammation in the context of NAS.

## Virulence and Host Association

In one study, the virulence potential of each *Staphylococcus* species and the profile of all Virulence Factors (VFs) were determined by defining a species-specific VF gene set from each species and analyzing variation within them (7). Virulence genes may explain why some species are more successful at colonizing and surviving within the udder, and products of such genes are considered VFs (7). The phylogenetic distribution, sharing and evolution of VFs can reveal how these different species evolved (7). Accordingly, if some NAS are commensals, a question of interest would be whether they individually became commensals or if they evolved from a common commensal ancestor. If the latter is true, they would have become more aggressive in claiming niches by accumulating VFs, leading to their evolution into a different species (7).

The distribution of 191 VFs and their possible associations with pathogenesis in 25 NAS species were determined along with the relationship between VFs and udder health (high SCC and signs of CM) (7). The overall number of VFs was not associated with disease severity. This confirmed data from another study in which virulence gene profile or accumulation of virulence genes did not predict the type of mastitis (SCM or CM) or the severity of inflammation (78). In one study, more severe disease outcomes were correlated with increasing numbers of toxin and host immune evasion genes (7). Although the effects of individual VFs have been analyzed (Table 3), these findings suggest that development of disease and interactions of VFs with the host are complex and determined by interplay of genes rather than just presence of specific virulence genes. Interactions of VFs expressed by these genes with the host could also depend on the specific staphylococcal species. Some NAS strains associated with mastitis had varying proportions of virulence genes, and biofilm formation genes were only detected in a small percentage of examined species (58). The contribution of virulence genes on disease outcomes or development can also be affected by intrinsic factors (within the udder) or extrinsic factors (in the cow's environment) that influence gene expression. The latter is likely influenced by factors such as herd management, climatic conditions, and geographic location. One study using NAS isolates from a single Chinese herd reported lower prevalence of exotoxin and biofilm-associated genes compared to previous studies (57). These findings suggest the need for additional studies on presence or absence of these genes, and further gene expression studies to resolve which are associated with disease severity. The lack of expression studies prevents us from understanding associations between specific NAS species and NAS IMI, as well as which genetic elements are responsible for differences in prevalence and distribution among NAS species. Additionally, molecular characterization resistance and virulence factors have also been conducted for small ruminant NAS (66). While there may be opportunities to learn from these studies, an important consideration is the potentially different host-pathogen interactions between cattle and small ruminants.

**Table 3:** Summary of virulence factors and their related genes that were detected in several NAS species, as well as the relationship between these genes and pathogenesis in the context of NAS IMI.

**Virulence factors (related genes)**	**Associations of virulence factors with pathogenesis of NAS**	**Reference(s)**
Methicillin-resistance and biofilm-related genes (*mecA, eno*)	• Isolates from clinical mastitis cases had a significantly higher presence of methicillin-resistant (*mecA*) genes (21 out of 43 isolates) • All 43 isolates tested positive for the presence of the biofilm-related gene, *eno*	(16)
Intracellular adhesin (*icaA/B/C*)	• In human-associated NAS, it is a genetic determinant for biofilm formation • Presence of *icaA* was associated with greater biofilm formation in bovine NAS species. Almost half the isolates tested positive for this gene	(7, 79, 80)
Iron-regulated surface determinant (*isdA/B/C/I*)	• *IsdI* the most frequently distributed gene among NAS species in a Canadian study • Every NAS isolate contained at least one gene related to iron uptake and metabolism • Staphylococci require iron to replicate and persist in infections	(7)
Hemolysin (*hla/b/d)*	• Hemolysins lysed erythrocytes of cattle, sheep, and goats • β-hemolysin (*hlb*) was the most frequent gene in NAS isolates in a Canadian study • In Iran, bovine NAS isolates primarily produced δ-hemolysin (*hld*)	(7, 81)
Phenol-soluble modulins (PSMβ1/2/3/4)	• Lysis of red and white blood cells, linked to biofilm formation and stimulation of inflammatory responses • β-type PSMs were associated with bovine NAS isolates in a Canadian study	(7)

**Figure 2:**
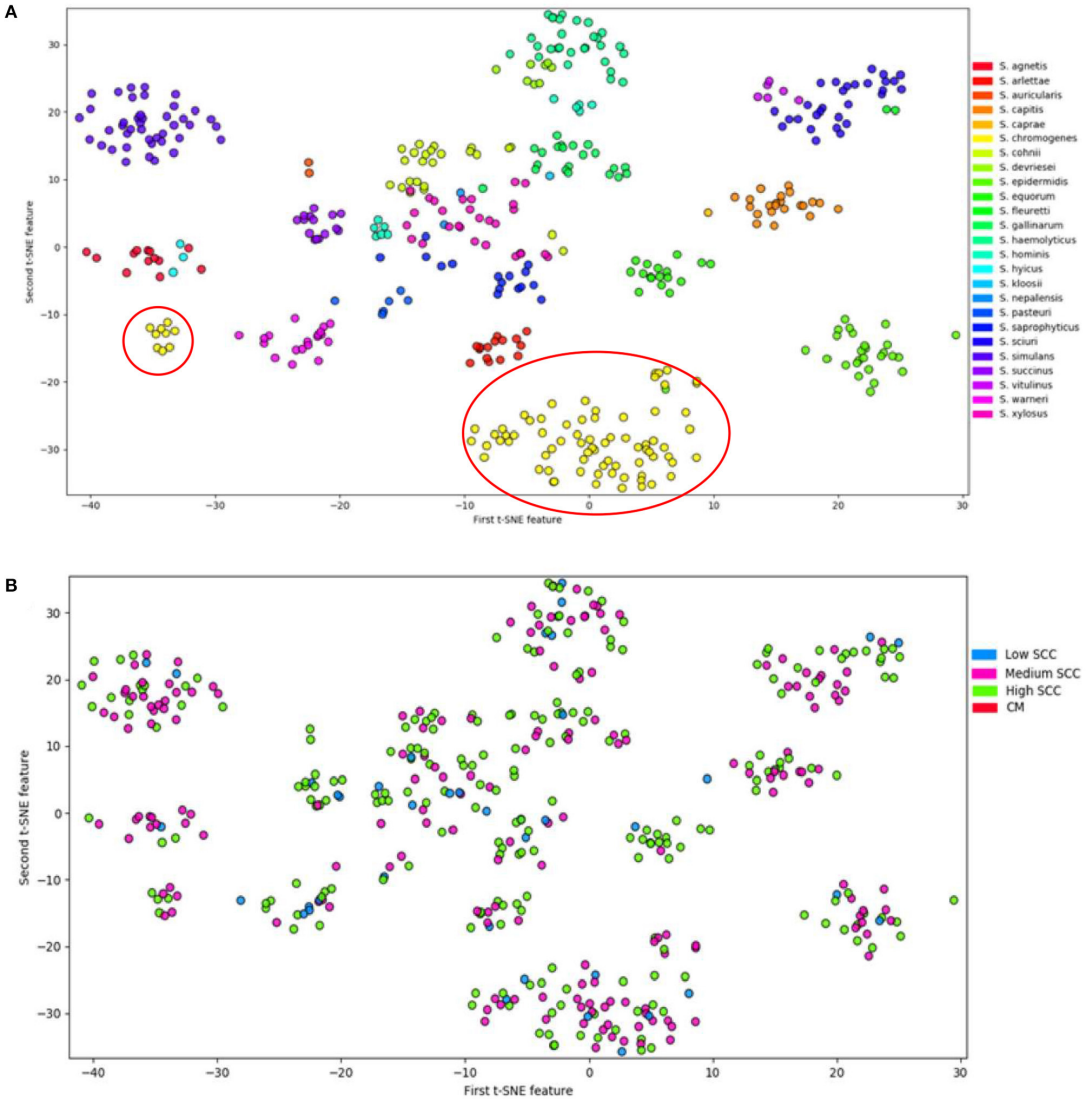
Visualization of 25 NAS species by t-Distributed Stochastic Neighbor Embedding (T-SNE) based on virulence gene content, demonstrating that each of the NAS species is a discrete microorganism; the red circles indicate the distinctive split between *S. chromogenes* populations **(A)**. Labeling of severity of mastitis of the NAS isolates separated based on virulence genes **(B)**. Modified from (7).

Analyses of the distribution of 191 VFs in 441 genomes of 25 NAS species by t-Distributed Stochastic Neighbor Embedding (T-SNE), a method to visualize high-dimensional datasets, demonstrated that all species studied can be defined as separate and homogenous bacteria (7) because of clear clustering by species (Figure 2). Virulence potential was also associated with the different phylogenetic clades. These findings suggest that virulence potential developed gradually during evolution into distinct species. This is in contrast to the possibility that some species acquired several virulence factors relatively suddenly, turning them into somewhat more virulent pathogens or more adapted commensals.

As discussed above, it is unclear what mechanisms enable *S. chromogenes* to be the most prevalent organism in bovine mastitis (and successful in causing persistent IMI and SCM). In-depth studies on genomes of 440 NAS isolates determined that closely related *S. chromogenes, S. agnetis*, and *S. hyicus* had the highest virulence potential (i.e., number of virulence genes), largely due to exotoxin, host evasion and capsular genes, of all NAS (82). However, *S. chromogenes* (~50% of NAS isolates) did not differ greatly in VF profile from the closely related species *S. agnetis* (<0.5% of NAS isolates) and *S. hyicus* (<0.1% of NAS isolates) (9). The lack of clear differences in detected virulence genes between *S. chromogenes* and the other Clade B NAS, despite the large differences in species distribution, suggest that an unknown mechanism is at play which makes *S. chromogenes* the most frequently isolated species in NAS IMI.

Interestingly, in the T-SNE plot (Figure 2), *S. chromogenes* is the only species split into 2 populations with respect to virulence genes, with a minority of the strains clustering with other members of the clade B, while the majority of the *S. chromogenes* strains have a distinct profile. An important caveat is that more *S. chromogenes* isolates were included in this study than other species, but it is tempting to speculate that the larger population of *S. chromogenes* might represent a pathotype that has adapted to the udder. Additional evidence for this was presented in a study demonstrating that *S. chromogenes* isolated from a chronic IMI had greater ability to adhere to bovine mammary epithelial cells compared to a strain isolated from the teat apex (83). Another study compared a *S. fleurettii* strain isolated from sawdust bedding and a *S. chromogenes* strain from a persistent IMI; the latter strain persisted longer after experimental inoculation into the udder (76). If true, the finding needs to be confirmed with a larger number of strains, as this might hold clues about why *S. chromogenes* has become the dominant NAS species isolated from milk of dairy cattle.

No clear difference was present between the two *S. chromogenes* populations with respect to severity of mastitis (Figure 2B). The subsequent sections will analyze how virulence factors may explain why *S. chromogenes* is the only species that diverges into two distinct populations. It is also important to note that other reasons may include differences in AMR profiles, host adaptation, interactions with host genetics and interactions with the microbiome.

In some *S. chromogenes* isolates capsular genes from the larger VF-based cluster are missing (7), which seems to be one factor that causes the population split in this species. In *S. aureus*, expression of these genes results in formation of a polysaccharide capsule that helps resist phagocytic cell uptake, thus playing a role in evasion of the host immune response (84). However, there is conflicting evidence on the associations between capsule genes and overall virulence of *Staphylococcus* species. In one study, presence of these genes and formation of polysaccharide capsules enhanced *S. aureus* virulence in a murine model, but decreased virulence of *S. aureus* when causing IMI (7). Based on a strong association between the amount of biofilm formed and the capsular genotype and phenotype, these factors may be important to virulence of *S. aureus* and its ability to persist in chronic IMIs. In a Canadian study, biofilm formation had no effect on disease severity (79). However, it has been suggested that biofilms increase the ability of NAS to persist in the mammary environment (79, 80). When analyzing *S. aureus* isolates *in vitro*, isolates which harbored genes coding for capsule type 5 (*cap5)* formed more biofilm and produced a thinner capsular polysaccharide layer than those with genes coding for capsule type 8 (*cap8)* (85). *S. chromogenes* isolates had *cap5* but *cap8* was not present (78). Additional *in vivo* testing is needed to better characterize the associations between pathogenicity and biofilm production in *S. chromogenes*.

Conversely, the absence of these capsular genes increased both intracellular survival rates as well as invasion rates of *S. aureus* (86). Persistence of this pathogen in an infected host has been linked to the loss of capsular polysaccharide 5 and 8 (CP5/8). This was confirmed in a murine model where isogenic acapsulated mutants persisted for a longer period of time and in higher numbers when compared to their capsulated counterparts (87). In clinical studies, human patients with chronic osteomyelitis had a higher proportion of non-typeable (NT) *S. aureus*, compared to those with acute osteomyelitis (87). NT strains are non-reactive with antibodies to CP types 1, 2, 5, or 8 (87), and these isolates from chronically infected hosts were shown to have conserved their acapsulated phenotype over successive passages on artificial media without reverting back to encapsulation (87). Isolates from cows with SCM revealed that the proportion of non-typeable (NT) *S. aureus* strains was 86% (88). These findings reveal that ability to persist in chronic infections is strongly associated with NT strains (i.e., acapsulated pathogens). With a majority of *S. chromogenes* isolates lacking capsule genes, it may be of further interest to study the relationship between acapsulation and the persistence of *S. chromogenes* in IMI.

Other previously identified VFs associated with pathogenicity of *S. aureus* have also been detected in NAS. β-hemolysin (*hlb*) was the most frequent and predominant gene detected in *S. chromogenes* isolates and other species of clade B (7). The *hlb* gene was detected in all isolates in clade D3, while only a few of the isolates in one clade E species carried this gene (7). Strains of *S. aureus* isolated from bovine CM produced predominantly β-hemolysin, in combination with other hemolysins (89). This was confirmed in a study which found that 97% of *S. aureus* isolates from Europe and the US either produced or were PCR-positive for β-hemolysin (90). It was also determined that 45–90 CFU of a β-hemolytic *S. aureus* strain could result in CM (89). These findings suggest that β-hemolysins may play an important role in the pathogenesis of mastitis caused by some strains of *S. aureus* but that it is not the sole virulence factor that influences disease severity. With almost all clade B isolates expressing the *hlb* gene (7), it may be of interest to elucidate its role in the pathogenesis of these species.

Adenosine synthase A was another *S. aureus* virulence gene detected in *S. chromogenes*. Adenosine synthase A is an immune evasion factor for *S. aureus* responsible for increasing the overall abundance of extracellular adenosine, which may be the most potent immuno-suppressive signaling molecule. This factor is necessary for staphylococcal survival within neutrophils, allowing *S. aureus* to escape bactericidal activity of leukocytes and other host immune responses (91, 92).

In the same study analyzing VF genes, all NAS species contained at least one gene from the iron-responsive surface determinant (*isd*) operon (7). Staphylococci require iron to replicate and sustain infections, and it was shown that the *isdI* gene, the most frequently distributed *isd* gene among all NAS species in this study, is necessary for *S. aureus* pathogenesis (7). Similarly, most NAS species contained β-type phenol-soluble modulins (PSMs), which have been considered major determinants of *S. aureus* virulence (7). Phenol-soluble modulins have multiple roles in staphylococcal pathogenesis, causing lysis in red and white blood cells, contributing to biofilm development and stimulation of inflammatory responses (93) (Table 3). The numerous studies above have studied the roles of these VFs in the pathogenesis of *S. aureus*. Perhaps elucidating their role in pathogenesis of *S. chromogenes* in future studies may explain its dominance in bovine mastitis and persistence in the udder (Table 3).

A correlation was observed between the average SCC of milk samples from which specific NAS species were isolated and the number of exoenzyme, host evasion and iron uptake genes these species carried (7, 9). These virulence genes might hold the key to why certain NAS species provoke somewhat more inflammation than others. Absence of these virulence genes may result in NAS species becoming more host adapted or even commensal. This is somewhat illustrated by *S. chromogenes*, which is considered a host-adapted NAS, and has moderate numbers of exoenzyme, host evasion and iron uptake genes. Furthermore, interesting associations were found between virulence genes identified in NAS, with striking differences in the strength of these associations between isolates that caused low SCC and CM isolates (7).

## Antimicrobial Resistance

The high prevalence of *S. chromogenes* relative to other NAS species is likely multifactorial. Antimicrobial resistance (AMR) might be one explanation for the predominance of a single species of *Staphylococcus* associated with the udder, but based on available data this does not seem to be the case. *S. chromogenes* have relatively low phenotypic and genotypic prevalence of AMR when compared to other NAS species isolated from the udder (82). Another study demonstrated that *S. epidermidis* had increased resistance rates against penicillin when compared to *S. chromogenes* (94). Additionally, researchers reported presence of β-lactamase in *S. chromogenes* is relatively low when compared to either *S. haemolyticus* or *S. epidermidis* (61).

In Canada, higher numbers of AMR genes, with a strong correlation between AMR genotype and phenotype, were identified in NAS rather than *S. aureus* originating from the same dairy herds (10, 82). This is in agreement with previous reports where *S. aureus* isolated from SCM and CM cases were less resistant than NAS against commonly used antimicrobials (60, 75, 95, 96). In addition, studies have demonstrated that NAS could serve as reservoirs for AMR genes for major mastitis pathogens including *S. aureus* (97, 98).

A study (60) investigated the association between AMR and antimicrobial use in NAS. An association was present when penicillins, third-generation cephalosporins or macrolides were administered systemically, but not when antimicrobials were administered via the intrauterine and intramammary route (99). It was hypothesized that antimicrobials administered systemically for conditions other than mastitis, if partitioning to the udder, could cause prolonged bacterial exposure to sub-therapeutic antimicrobial concentrations in the udder. Similarly, one study suggested that increasing systemic administration resulted in a decrease of antimicrobial susceptibility of NAS to β-lactams, as opposed to intramammary treatment of SCM. Systemic administration was expressed as antimicrobial treatment incidence, with units of the number of defined daily doses animal used per 1,000 cow-days (100). In addition, there is a higher likelihood of NAS being present in the udder compared *S. aureus*, which would therefore result in an increased window of exposure of NAS to antimicrobials used in dairy herds.

Methicillin-resistant NAS were an important reservoir of AMR and virulence genes in a Belgian study (98). Most cases saw an association between presence of AMR genes and phenotypic resistance, and only a few cases had a negative correlation between presence of AMR genes and resistance. This study also identified some isolates which did not carry any of the investigated AMR genes yet still displayed a non-wild type (epidemiologically resistant) phenotype (98). *Staphylococcus sciuri* appeared resistant to fusidic acid but this phenotype was not correlated to any of the known fusidic acid resistance genes (98) (Table 4). In a Swiss study (98) *in vitro* phenotypic resistance to several antimicrobials such as erythromycin, clindamycin and streptomycin was not explained by the presence of any tested genes (102), suggesting development of new resistance mechanisms. Previous studies have also characterized associations between resistance determinants and AMR in NAS. These include β-lactam resistance being associated with *blaZ* and *mecA* genes, and chloramphenicol resistance having a correlation with the FexA transporter. Daptomycin resistance was explained by the presence of the *mprF* gene, whereas *tetK* and *tetL* genes were associated with tetracycline resistance (82). Even though bovine NAS isolates may acquire resistance to these antimicrobials, it has been suggested through phenotypic AMR patterns that intrinsic mechanisms of AMR may be present for a subset of NAS species as well (82). It is worth noting that many of these antimicrobials are not labeled for use in lactating dairy cows. For example in North America and Europe chloramphenicol is illegal for use in food-producing animals, suggesting the absence of selective pressures.

**Table 4:** Summary of antimicrobial resistance profiles and prevalence of single gene resistance determinants of frequently isolated NAS species across several studies.

**Species**	**Antimicrobial resistance profile^*a*^**	**Resistance determinants (% prevalence of determinant)**		**References**
		**Acquired**	**Intrinsic**	
*S. lentus*	PEN, KAN, STR, TET, CHL, TMP, FUS	*tetK (23), tetM (10), tetL (14), fexA (32)*	–	
*S. sciuri*	PEN, FOX, GEN, KAN, STR, ERY, CLI, SYN, TET, CHL, TMP, FUS	*tetK (5), tetL (25), fexA (5)*	–	(98)
*S. epidermidis*	PEN, FOX, GEN, KAN, STR, ERY, CLI, TET, CHL, TMP, FUS	*tetK (45), tetL (9), fexA (27)*	–	
*S. epidermidis*	NA, ERY, KAN, GEN, TOB	–	–	
*S. simulans*	NA, ERY, STR, CLI	–	–	(101)
*S. haemolyticus*	NA, SXT	–	–	
*S. chromogenes*	NA, TET	–	–	
*S. chromogenes*	PEN, OXA, STR, TET, ERY	*blaZ (72), mecA (2)*	–	
*S. epidermidis*	PEN, OXA STR, TET, ERY	*blaZ (48), mecA (4)*	–	(94)
*S. xylosus*	PEN, OXA, STR, ERY	*blaZ (87)*	–	
*S. chromogenes*	PEN, OXA, TET, STR, ERY, CLI, CHL, KANA, GEN, TRM	–	–	
*S. xylosus*	PEN, OXA, TET, ERY, CLI, CHL, GEN	–	–	(102)
*S. sciuri*	OXA, TET, STR, CLI, KAN, GEN, TRM	–	–	
*S. chromogenes*	CHL, TET, CLI, PNV, PIR, ERY, AMP, PEN	*blaZ (10), tet38 (100), tetK (2), tetL (3)*,	*NorA (100), Sav1866 (100)*,	
*S. simulans*	CHL, TET, PIR, ERY, PEN, MDR	*tetK (3), tetL (3), tetM (3)*	*norA* (100), *Sav1886* (100).	(82)
*S. xylosus*	CHL, TET, CLI, PIR, ERY, AMP, PEN, MDR	*msrA (14), tetK (19)*	*norA (100), norB (100), sav1886 (100)*	
*S. arlettae*	–	–	*bla_*ARL*_*	(103)

*Isolates from bovine milk diagnosed with clinical and subclinical mastitis were used in all studies with the exception of the first study which used nasal swab samples collected from veal calves*.

a *Multi-drug resistant profiles are not included. NA, nalidixic acid; ERY, erythromycin; KAN, kanamycin; GEN, gentamicin; TOB, tobramycin; STR, streptomycin; SXT, sulphamethoxazole-trimethoprim; TET, tetracycline'; PEN, penicillin; CLI, clindamycin; CHL, chloramphenicol; TRM, trimethoprim; FUS, fusidic acid; FOX; OXA, oxacillin; PIR, pirlimycin*.

A study in Portugal characterized the AMR profile of methicillin-resistant staphylococci (MRS) isolates from bovine SCM and CM cases, identifying 9.3% of isolates as being MRS and associated with the *mecA* virulence gene (101). Despite the low percentage of MRS detected, the majority of isolates still had a multi-resistance profile (101) (Table 4). This study, in addition to a Swedish one (61), revealed that AMR and virulence gene profiles are species dependent. The Swedish study revealed that the prevalence of β-lactamase varied among NAS species and was more common in isolates originating from SCM cases than from CM cases (61). β-lactamase is the most common resistance mechanism in staphylococci, and while the prevalence was high in *S. epidermis* and *S. haemolyticus*, there was little to no detection in *S. chromogenes* and *S. simulans* (61). In this study, *S. chromogenes* and *S. epidermis* were the most commonly isolated species in SCM cases (61). In a Dutch study, 70% of *S. epidermis* isolates and 18% of *S. chromogenes* isolates were resistant to penicillin (94) (Table 4), suggesting that a high prevalence of penicillin resistance in SCM was associated with the high prevalence of *S. epidermis* (61). These findings confirm the existence of inter-species variation in AMR profiles, emphasizing the need to continue monitoring co-resistance profiles among NAS populations associated with bovine mastitis cases. Coupled with the possible development of resistance mechanisms not associated with previously characterized virulence genes, additional studies analyzing AMR in NAS are needed alongside the characterization of bovine NAS specific clinical antimicrobial susceptibility breakpoints, as this presents a challenge in treating bovine mastitis cases.

## Niche Adaptation and Host Association

NAS prevalence and distribution is impacted by many environmental and management factors such as geographic region, climate, water sources, access to pasture, barn type, bedding and host factors (parity, quarter location, antibiotic use). In this context, it is useful to determine the natural habitat of different NAS species. This defines whether they should be considered as environmental or host-adapted pathogens. This also relates to their commensal nature and their level of host adaptation to the skin, teat canal and/or udder.

Host adaptation relates to colonization and persistence of isolates as well as the level of inflammation caused. Adaptation can be quite specific, demonstrated by the fact that species and frequency of isolation of NAS differs between teat canal and milk samples (104). Some studies find the most predominant NAS species, *S. chromogenes* and *S. xylosus*, to be equally ubiquitous in CM, SCM, skin, and environment (61, 75). These two species are also more frequently associated with persistent IMI and SCM compared to other NAS species (55). Other studies report differences in distribution and in genotypes between milk, udder and environment (60, 105). In contrast, molecular epidemiology studies demonstrate that *S. haemolyticus, S. fleurettii*, and *S. equorum* are predominantly environmental species (55, 105).

It was clearly demonstrated that some NAS species are more associated with IMI than with environmental (e.g., parlor-associated) niches (105). Interestingly, *S. chromogenes* is almost uniquely associated with IMI and not found in the environment of the dairy cow. Unpublished data from Walpole et al. comparing isolates identified in milk vs. body sites, failed to detect *S. chromogenes* a single time on other body sites of dairy cattle, whereas it was by far the most frequently isolated species from IMI. In contrast, Adkins et al. (44) isolated *S. chromogenes* from pre-partum mammary secretions, milk, the inguinal region skin, teat skin muzzle, and perineum of peripartum dairy heifers. Hence, these data demonstrate likely adaption to niches on the cow which seems to underpin its success as an IMI organism. Similar data have been reported for *S. aureus*, another host-adapted udder pathogen (106).

A recent longitudinal study, identified 4 udder-adapted NAS species, 2 of which were considered persistent and some demonstrated characteristics of contagious pathogens (*S. chromogenes* and *S. simulans*) (55). Contagious transmission routes for *S. chromogenes* and *S. simulans* seem plausible from this study, possibly in addition to environmental transmission patterns.

## Interactions Within the Microbiome

Knowledge is emerging that an udder microbiome exists that is distributed over the milk, milk ducts, cistern, teat canal, teat apex and teat skin, where staphylococci seem to play an important role (12). Previous literature used the NMC procedure to define NAS-positive samples (Figure 2), importantly at the time of publication, the National Mastitis Council (NMC) did not publish guidelines for classifying quarters as infected or not in the context of clinical diagnosis for IMI (107). Another consideration is that culture-independent genotypic methods have only been implemented during the past decade for use in IMI diagnosis (108). The bovine milk microbiome in both culture-based and DNA-based methods has proven to be more complex than expected, with the role of different species in milk samples—either as pathogens, commensals, or contaminants—being essential in assessing the analyses (108). Currently, the best definition of IMI is offered Dohoo et al. (107), but even these criteria can be open to interpretation. *S. chromogenes* is one of the organisms most negatively influencing the microbiome of the udder based on the observation that it has the most negative connections with other members of the milk microbiota. These negative connections presumably reduce diversity and therefore microbiome stability (109). A similar negative effect was observed for *S. xylosus*. In general, staphylococci are negatively correlated with Shannon and Simpson indices of diversity (109). Conflicting evidence exists on whether or not they are disruptors of the normal milk microbiome (12, 109). The negative interactions might be due to indirect mechanisms that involve the host, such as the induction of immune responses, or may be due to other genera in the microbiome that are overshadowed by NAS.

Direct mechanisms, including the production of antimicrobial factors such as bacteriocins, may also result in negative correlation between *S. chromogenes* and other members of the milk microbiota. NAS produce many of these bacteriocins with capacity to inhibit the growth of mostly Gram-positive bacteria but also some with potential to inhibit Gram-negatives (110). A Belgian study found that 38 of 254 NAS isolates displayed bacteriocin-like activity, and that 7 of these strains displayed activity against at least one major pathogen associated with bovine mastitis (111). Interestingly, the bacteriocin produced by an inhibitory *S. chromogenes* strain used in this study (nukacin L217) inhibited the growth of all mastitis-causing pathogens tested (111). This bacteriocin may hold clues to the success of *S. chromogenes* as an NAS species in IMI and its possibly negative associations with major mastitis pathogens such as *S. aureus*, as antibacterial production is often advantageous for strain colonization in a certain niche (111). These findings are mostly based on *in vitro* studies. It remains unclear if these bacteriocins play an actual role in modulating the microbiota inside the udder or on the skin, as in the Belgian study bacteriocin production was abundant on growth agar medium but did not grow in broth (111). Other species apart from *S. chromogenes* also inhibit the growth of major mastitis pathogens. In a recent study, cytoplasmic bacteriocins from *S. epidermidis* selectively inhibited growth of *S. aureus*, including methicillin-resistant strains (112). These studies suggest the need for additional *in vivo* studies to determine how bacteriocins influence NAS species-level interactions in the milk microbiome.

Previous studies have clearly established that co-infections with other NAS and pathogens occur (113–115), yet there exists conflicting evidence as to whether NAS increase susceptibility to major pathogens such as *S. aureus* or prevent it from colonizing the udder. Because major pathogens are generally considered more virulent and damaging to the udder than minor mastitis pathogens such as NAS, it would be of interest to clarify what impact NAS has on major pathogens. Several studies detailed analyses which concluded that NAS colonization protected quarters against IMI by major pathogens (88, 116, 117), whereas another reported that the presence of NAS was a risk factor for acquiring *S. aureus* IMI (118). Interestingly, certain strains of *S. chromogenes* can inhibit the *in vitro* growth of all *S. aureus, S. dysgalactiae*, and *S. uberis* strains. The intensity of inhibition varied amongst target species, with only 2 out of 10 *S. chromogenes* isolates showing consistent inhibitory activity (117). A systematic review of the current literature revealed that strong protective effects were observed in studies that had higher underlying risks, as well as in challenge studies which introduced major pathogens into the udder through the teat end (113). Studies that used larger doses of challenge organisms and those with more stringent diagnostic criteria for pathogen IMI reported reduced protective effects. Larger scale studies are needed to resolve the existing conflicting evidence and better characterize the association between NAS and major pathogens.

Interestingly, there also seems to be a host genetic component to whether NAS are part of the milk microbiome. Two main variants of the bovine antigen presenting major histocompatibility complex protein Bola DrB3.2 strongly defined what organisms are “accepted” to form the milk microbiome (119). Each of the genetic variants seems to promote the presence of different NAS species: *S*. *equorum, S. gallinarum, S. sciuri*, and *S*. *haemolyticus* were enriched in microbiota of one of the variants, whereas *S*. *chromogenes* was enriched within microbiota of the second variant. These findings spark hypotheses related to the predominance of *S. chromogenes* and the dichotomy between “environmental” and “host adapted” NAS.

## Conclusions

**Figure 3:**
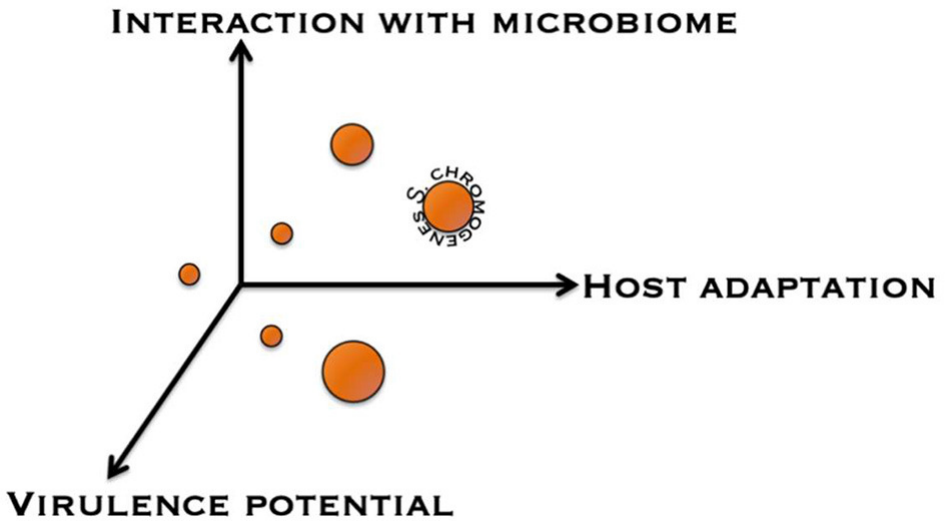
Conceptual discrimination of different non-*aureus* staphylococci based on several factors. Displayed are the interaction with the udder microbiome, host adaptation, and virulence potential.

To help improve our understanding as to whether NAS species are commensals, opportunistic pathogens, or obligate (minor) pathogens with respect to the udder, a framework was conceptualized to categorize NAS based on different discriminating factors (Figure 3). A first factor is the nature of the interaction the NAS species has with the udder, ranging from a commensal interaction to a pathogenic interaction. A second factor is the strength and specialization behind this interaction, from environmental organism to obligate symbiont. A third factor is the impact of the NAS species on the milk microbiome and on major mastitis pathogens. NAS make up a significant fraction of the milk microbiome (109) and they also seem to contribute to many of the predicted interactions between milk microbiome members. This categorization might help in defining which NAS dairy producers should consider more important than others when designing control programs. Additional factors could include antimicrobial resistance and compatibility with host immune genetics and response.

Although many recent studies have focused on NAS at the species level, many questions remain (Table 1). The true nature of each NAS species has yet to be identified, either as commensals or pathogens, or as environmental or contagious pathogens. The effects of these interactions between NAS with the rest of the milk microbiome as well as its associations with host genetics and the immune response need to be elucidated. Interactions in the milk microbiome may influence factors such as AMR or virulence in NAS species, leading to their success as colonizers of the udder. Further investigations into the role of NAS as an AMR reservoir for major and minor pathogens are needed. In addition, more data is needed to clarify if NAS truly prevent other mastitis pathogens from colonizing or infecting the udder.

It will also be worthwhile to elucidate the reason for dominance of *S. chromogenes* with the NAS in many parts of the world. This is particularly important as it is unclear if *S. chromogenes* should be considered beneficial or harmful. Given *S. chromogenes'* dominance as NAS and IMI commensal or pathogen in general and its potentially positive or negative impacts, it seems that new strategies to support or eliminate *S. chromogenes* from the bovine udder would go a long way in reducing the prevalence and impact of mastitis in dairy herds. It should be determined which other NAS species have the same impact as *S. chromogenes*, in addition to which species require less or no attention, as NAS may represent a natural mechanism to reduce IMI with (other) mastitis pathogens, which could be implemented as an intervention method. It is also important to focus on strain differences related to interactions of NAS with the udder, as they may override differences at the species level.

Finally, for herds that have successfully controlled other mastitis pathogens, controlling cases of NAS CM and SCM may be an important step in further lowering bulk milk SCC. While much has been reported to help define the ecology and epidemiology of these bacteria, a clear understanding of how existing mastitis control practices can be applied or where new control measures are needed to mitigate IMI is needed.

## Author Contributions

JD, VH, SN, DN, CL, JM, SD, and HB contributed to writing and editing the review. All authors contributed to the article and approved the submitted version.

## Conflict of Interest

The authors declare that the research was conducted in the absence of any commercial or financial relationships that could be construed as a potential conflict of interest.
